# High spatial resolution time-resolved magnetic resonance angiography of lower extremity tumors at 3T: Comparison with computed tomography angiography

**DOI:** 10.1097/MD.0000000000004894

**Published:** 2016-09-16

**Authors:** Gang Wu, Teng Jin, Ting Li, John Morelli, Xiaoming Li

**Affiliations:** a Department of Radiology, Tongji Hospital, Tongji Medical College, Huazhong University of Science and Technology; b Department of Radiology, Wuhan NO.1 Hospital, Wuhan, Hubei, China; c St John Medical Center, Tulsa, OK..

**Keywords:** lower extremity, magnetic resonance angiography, time-resolved, tumors

## Abstract

The aim of this study was to compare diagnostic value of high spatial resolution time-resolved magnetic resonance angiography with interleaved stochastic trajectory (TWIST) using Gadobutrol to Computed tomography angiography (CTA) for preoperative evaluation of lower extremity tumors.

This prospective study was approved by the institutional review board. Fifty consecutive patients (31 men, 19 women, age range 18–80 years, average age 42.7 years) with lower extremity tumors underwent TWIST magnetic resonance angiography (MRA) and CTA. Digital subtraction angiography was available for 8 patients. Image quality of MRA was compared with CTA by 2 radiologists according to a 4-point Likert scale. Arterial involvement by tumor was compared using kappa test between MRA and CTA. The ability to identify feeding arteries and arterio-venous fistulae (AVF) was compared using Wilcoxon signed rank test and McNemar test, respectively.

Image quality of MRA and CTA was rated without a statistically significant difference (3.88 ± 0.37 vs. 3.97 ± 0.16, *P* = 0.135). Intramodality agreement was high for the identification of arterial invasion (kappa = 0.806 ± 0.073 for Reader 1, kappa = 0.805 ± 0.073 for Reader 2). Readers found AVF in 27 of 50 MRA cases and 14 of 50 CTA cases (*P* < 0.001). Mean feeding arteries identified with MRA were significantly more than that with CTA (2.08 ± 1.72 vs. 1.62 ± 1.52, *P* = .02).

TWIST MRA is a reliable imaging modality for the assessment of lower extremity tumors. TWIST MRA is comparable to CTA for the identification of AVF and feeding arteries.

## 1 Introduction

Needle biopsy before surgical resection is widely accepted for assessment of lower extremity tumors.^[1]^ However, preoperative imaging tests remain indispensable, even when histological results were known because imaging of tumors assists to design surgical planning.^[2]^ Preoperative vascular evaluation of tumors can even alter the operative approach.^[3]^ Assessment of arterial invasion with imaging is helpful in deciding whether tumor could be surgically resected.^[4]^

Catheter digital subtraction angiography (DSA) is the diagnostic criterion standard for most vascular imaging owing to its high temporal and spatial resolution. Preoperative embolization can be performed concurrently in some cases to prevent hemorrhage during the definitive surgery. However, the performance of DSA is limited in practice, as it is an invasive and expensive test relying upon the use of ionizing radiation.^[5–7]^ Computed tomography angiography (CTA) and magnetic resonance angiography (MRA) are noninvasive alternatives to DSA for the depiction of tumors.^[8–11]^ With respect to MRA, time-resolved MRA has been reported as superior to conventional CE-MRA in several respects: lower contrast agent dose, decreased venous contamination, and added dynamic information.^[12–15]^ TWIST (Time-resolved magnetic resonance angiography With Interleaved Stochastic Trajectories, TWIST) imaging divided k-space into 2 regions: one central region responsible for overall image contrast and a peripheral, outer region responsible for image detail.^[16]^ To accomplish greater temporal resolution, the central regions are sampled more frequently than the peripheral regions based on a technique called view sharing. Although TWIST MRA has been reported to yield excellent image quality and to be accurate in the diagnosis of peripheral arterial stenosis,^[11]^ its value in the assessment of lower extremity tumors is beyond the authors’ knowledge. The aim of the present study is to compare TWIST MRA with CTA for the preoperative vascular evaluation of lower extremity tumors.

## 2 Material and methods

### 2.1 Patients

This prospective study was institutional review board-approved (IRB of Huazhong University, Wuhan, China). Inclusion criteria were: a patient with a lower extremity mass; tumor protocol MRI confirming presence of a mass; a surgical plan for open biopsy or definitive surgery; patient agreement to both CTA and MRA. Exclusion criteria were: patient’ glomerular filtration rate <30 mL/m^2^/1.73 m^2^; patient on dialysis; history of kidney or liver transplantation; pregnancy; standard contraindications for MR or CT (i.e., pacemaker, paramagnetic foreign bodies, claustrophobia, among others). From January 2013 to March 2015, 57 consecutive patients met the inclusion criteria and were enrolled in this trial. Informed consent was obtained from every patient. All 57 patients underwent TWIST MRA. Fifty-five of 57 patients underwent CTA, whereas 2 patients with contrast allergy did not undergo CTA. Five cases were excluded because no tumor was ultimately found; thus, 50 patients with lower extremity tumors were included in the final analysis.

### 2.2 MRA

All MRA examinations were performed on a 3.0T whole-body MR scanner (Magnetom Skyra, Siemens medical solutions, Germany). Patients were placed on the scanner table in feet-first supine position. Two eight-element body array coils were used to cover the area of interest, and were combined with the posterior integrated multichannel spine array coil. An 18-gauge intravenous line was placed in the right antecubital vein for contrast injection.

TWIST MRA was performed in the coronal plane with the following parameters: TR/TE, 2.9/1.06 ms; flip angle, 25°; bandwidth, 700 Hz; field of view (FOV), 448 mm × 358.4 mm; slice thickness, 1 mm; matrix, 450 × 360; spatial resolution, 1 mm × 1 mm × 1 mm; time resolution, 3.84 s/frame; A&B, 15%/20%; GeneRalized Autocalibrating Partially Parallel Acquisition (GRAPPA) factor, 2; number of measurements, 25.

Gadovist (Gadobutrol, Bayer Pharma AG, Berlin, Germany) was utilized as the contrast agent in this study (C_18_H_31_GdN_4_O_9_ in molecular formula, and 604.72 in molecular weight). Injection of contrast (0.1 mmol/kg body weight) was given by an automatic injector (Accutron MR, Medtron, Germany) at a rate of 2.5 mL/s, followed by a 20-mL saline flush at the same rate.

### 2.3 CTA

For each patient, the CTA examination was performed within 36 hours after MRA. A 128-row CT scanner (Discovery HD 750, GE medical) was used with the following parameters: tube voltage, 100 Kv; tube current, 150 mA; pitch, 0.984:1; table speed, 55 mm/s; slice thickness, 0.625 mm; FOV, 50 cm. Iodinated contrast agent (Ultravist, Bayer, Germany, 1.2 mL/kg body weight) was administered by an electronic power injector (Stellant, MEDRAD) through an 18-gauge antecubital injection at a rate of 3 mL/s. The bolus-tracking technique was used. A region of interest (ROI) was positioned in the aortic bifurcation and image acquisition automatically started 5.5 seconds after the signal attenuation of ROI reached the predefined threshold of 120 Hounsfield Units (HU).

### 2.4 DSA protocol

DSA correlation was available in 8 patients. For each patient, DSA was performed within 36 hours after CTA by an interventional radiologist with 18 years’ experience on a clinical DSA unit (Allura Xper FD20, Philips, Holland). 6 mL of iodinated contrast material (Ultravist, Bayer, Germany) was administered at the rate of 3 mL/s for each DSA run. Multiple runs were performed based on individual patient requirements at the discretion of the interventional radiologist. Preoperative gel foam embolization (Gelfoam, Alicon, Hangzhou, China) was performed in 5 of 8 patients.

### 2.5 Image analysis

Imaging post-processing was performed on a dedicated Siemens workstation (Syngo.Via). Two readers with 10 and 8 years’ experience viewed maximum intensity projections (MIPs) images of CTA and arterial phase MIP images of TWIST for image quality evaluation. MRA MIPs were generated automatically by the scanner. CTA MIP images were reconstructed with a window setting of 600/300 (window width/ window level). The readers were blinded to diagnosis and patient information and viewed cases in random order. The readers evaluated the MIP images according to a 4-point Likert scale: 0 = nondiagnostic image quality; 1 = poor image quality, observer not confident; 2 = fair quality, observer marginally confident; 3 = good quality, observer confident, 4 = excellent quality, observer highly confident. If the readers gave different scores for the same image, the average of the 2 scores was utilized for the analysis.

### 2.6 Arterial involvement

Invasion of the closest major arterial structures to the tumor was evaluated by 2 readers with 8 and 6 years’ experience. Both source images and reconstructed images were assessed. Arterial involvement was assessed as follows: 0—artery free from tumor; 1—artery displaced but not narrowed by tumor; 2—tumor narrowing artery <50%; 3—tumor narrowing artery ≥50%. The readers were blinded to diagnosis and patient information, and rated cases in random order.

### 2.7 Arteriovenous fistulae and feeding arteries

The presence or absence of arteriovenous fistulae (AVF) was determined by 2 readers with 10 and 9 years’ experience in consensus. A fluoroscopic TWIST MIP was examined to confirm the diagnosis of AVF. The 2 readers were asked to count feeding arteries of each case in consensus using MIP images. The viewing interval between CTA and MRA for the same patient was at least 3 weeks to avoid recall bias. The DSA images of 8 patients were reviewed by an interventional radiologist with 20 years of experience, who was blind to patient information and other imaging results (including CTA and MRA). The presence or absence of AVF in each patient was determined by the interventional radiologist, and the feeding arteries of each case were recorded.

### 2.8 Operation and pathology

**Figure 1:**
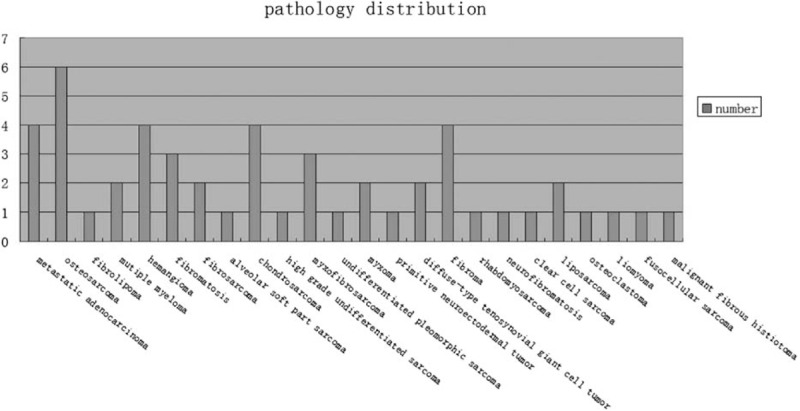
Distribution of histopathology for the lower extremity tumors of the 50 evaluated patients.

For each patient, a definitive operation or open biopsy was performed within 48 hours following the CTA examination. Mass resection after biopsy was performed in 32 cases. An associated joint replacement was performed in 3 cases. An amputation at the level of the right hip joint was performed in one patient. Fourteen patients received incision biopsy without mass resection. Pathological correlation was available in all 50 cases (Fig. 1). In the present study, WHO classification of tumors of soft tissue and bone 2013 (4th edition) was used.^[17]^ Thirty-eight of 50 cases were primary soft tissue tumors and 12 of 50 cases were bone tumors with soft tissue involvement. Eighteen cases were benign tumors, and 32 cases were malignant tumors.

### 2.9 Statistical analysis

The Wilcoxon signed rank test was used to determine differences in image quality of CTA and MRA, and also used to compare number of feeding arteries at CTA and MRA. Intramodality agreement in detecting arterial involvement was determined with a Kappa test. Kappa >0.8 was considered as excellent agreement; 0.6∼0.8 was considered good; 0.4∼0.6 was considered fair; Kappa <0.4 was considered as poor agreement. The percentage of AVF at CTA cases was compared with MRA cases using the McNemar test. All data analyses were performed with SPSS (version 21.0, IBM). A *P* value <0.05 was considered statistically significant.

## 3 Results

From January 2013 to March 2015, 50 consecutive patients (31 men, 19 women, age range 18–80 years, average age 42.7 years) with lower extremity tumors underwent TWIST MRA and CTA, without any adverse events.

**Figure 2:**
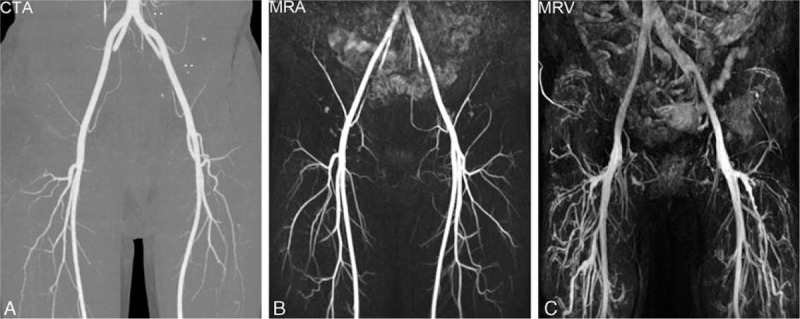
(A) CTA image rated as excellent image quality. (B) Arterial phase TWIST image rated as excellent image quality. The arterial SNR is high with small branches clearly depicted. (C) Venous phase TWIST image with diagnostic image quality and no arterial contamination. CTA = computed tomography angiography, MRA = magnetic resonance angiography, MRV = magnetic resonance venography.

Forty-four MRA cases and 48 CTA cases were rated as excellent by both readers (Fig. 2). Image quality of MRA was lower than that of CTA in 6 of 50 cases. Mean image quality of MRA was slightly lower than that of CTA, not reaching statistical significance (3.88 ± 0.37 vs. 3.97 ± 0.16, *P* = 0.135).

**Figure 4:**
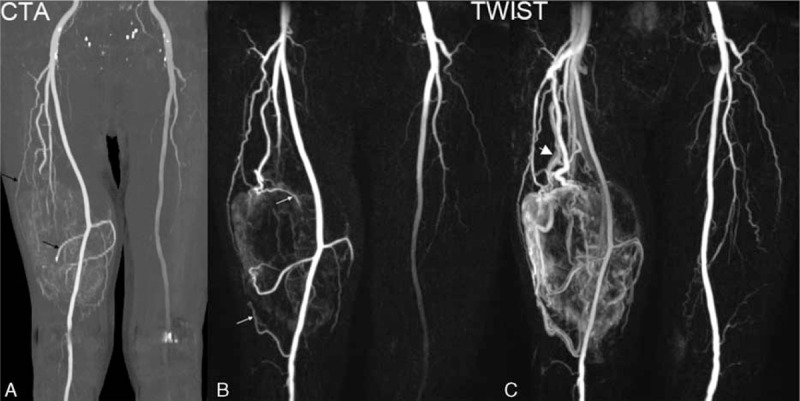
Malignant fibrous histiocytoma (A) CTA demonstrating the right superficial femoral artery displaced by a mass in the right distal thigh without evidence of stenosis. Increased number and abnormal morphology of small arterial branches are identified. Several feeding arteries (thin black arrows) and tumor stain are also demonstrated. (B) Right superficial femoral artery involvement with MRA was rated equivalent to that of the arterial involvement demonstrated by CTA. More feeding arteries (thin white arrows) are identified on MRA. The feeding arteries in MRA are more distinct than those identified by CTA. (C) Arteriovenous fistula (thick white arrow) and abnormal enhancing nidi are well-seen in the later phase of TWIST imaging. Most of the draining veins corresponded to the feeding arteries. CTA = computed tomography angiography, TWIST = time-resolved magnetic resonance angiography With Interleaved Stochastic Trajectories.

Invasion of the closest major arterial structure is shown in Table 1. Intramodality agreement between MRA and CTA (Table 2) in determining the involvement of arterial structures was excellent for both readers (kappa = 0.806 ± 0.073 and 0.805 ± 0.073). Eighty-eight percent cases (44/50) were rated equally with CTA and MRA (Fig. 4). In 3 cases, arterial involvement was assigned a score of 1 with MRA versus a score of 2 with CTA. In 2 cases, arterial involvement was assigned a score of 1 with MRA but 0 with CTA.

**Table 1:**
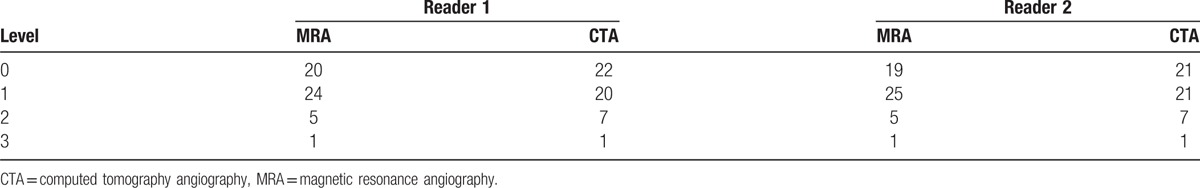
Involvement of the closest major arterial structure to the tumor was rated: 0—artery free from tumor; 1—artery displaced but not narrowed by tumor; 2—tumor narrowing artery less than 50%; 3—tumor narrowing artery ≥50%.

**Table 2:**
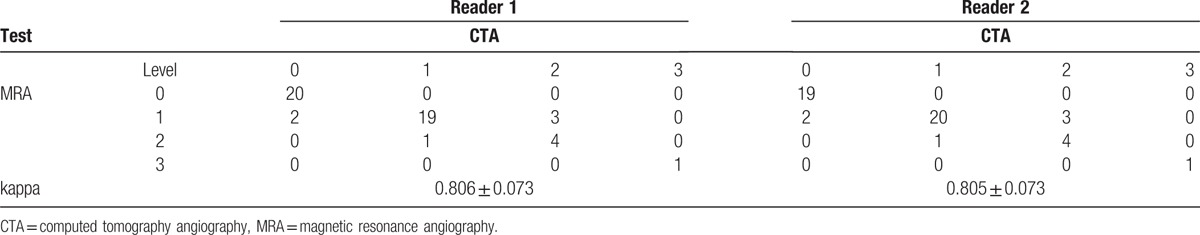
Agreement between MRA and CTA in determining arterial involvement.

**Figure 3:**
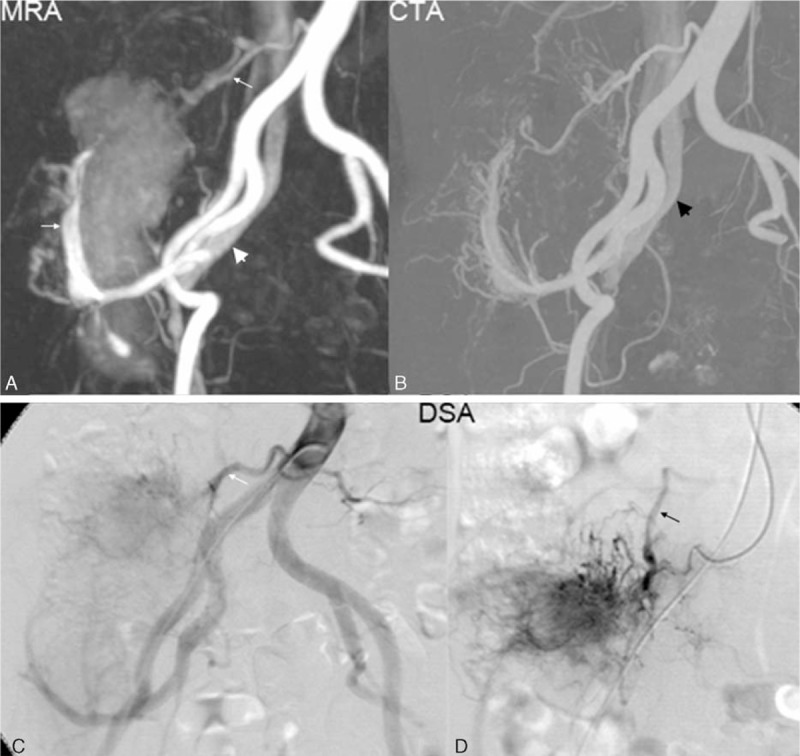
Multiple myeloma. (A) MRA of a tumor within the right thigh with contrast staining, 2 feeding arteries (thin white arrows), and an arterio-venous fistula (thick white arrow). (B) CTA with excellent depiction of arterio-venous fistula (thick black arrow) and feeding arteries. (C) Analogous findings as depicted on DSA. Here both feeding arteries (thin white arrow) are identified along with tumor stain from contrast administration. (D) Ultraselective DSA with the tip of the catheter in the right fourth lumbar artery. This artery was proven to be the feeding artery of the tumor. Subselectively, tumor stain was even more intense than in C. An abnormal early draining vein (thin black arrow) was prominent in D consistent with an arterio-venous fistula. CTA = computed tomography angiography, DSA = digital subtraction angiography, MRA = magnetic resonance angiography

**Figure 5:**
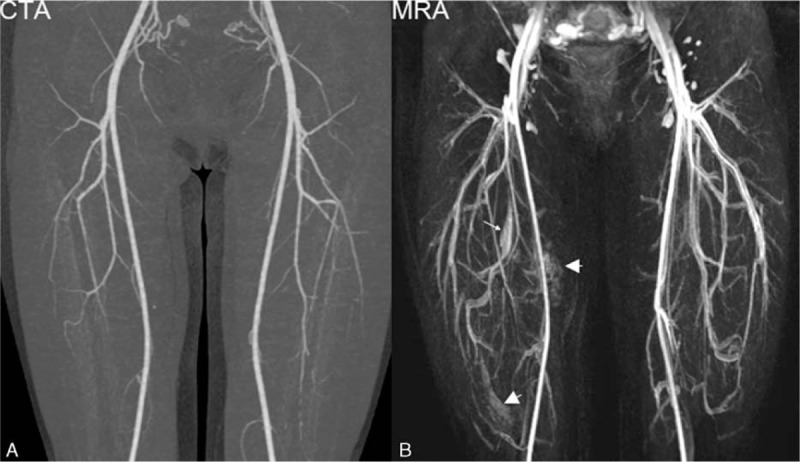
Intramuscular hemangioma. (A) An arterial abnormality is difficult to identify on the CTA MIP image. (B) Subtle perivascular enhancement (thick white arrows) and a dilated draining vein (thin white arrow) on MRA are consistent with the presence of a vascular malformation. CTA = computed tomography angiography, MRA = magnetic resonance angiography.

Readers identified AVF in 27 of 50 MRA cases, and in 14 of 50 CTA cases (54.0% vs. 28.0%, *P* < 0.001). MRA identified the same number of feeding arteries as CTA in 29 of 50 cases (Fig. 3), while identifying ≥1 feeding arteries than CTA in 18 of 50 cases (Fig. 4). The mean number of feeding arteries identified by MRA was greater than that of CTA (2.08 ± 1.72 vs. 1.62 ± 1.52, *P* = 0.02). In an intramuscular hemangioma case, TWIST identified perivascular enhancement and a dilated draining vein, which were not seen on CTA (Fig. 5).

For 8 patients, DSA correlation was available. Comparison between DSA, MRA, and CTA regarding the feeding arteries and AVF is shown in Table 3. DSA revealed 1 more feeding artery (right descending genicular artery) than MRA for a single patient (patient number 5). For 7 patients, the identity and number of feeding arteries identified by MRA and DSA were equivalent (see Table 3). MRA revealed 1 more feeding artery (right lateral femoral circumflex artery) than CTA for 1 patient (patient number 3). AVF were detected by MRA and DSA in 4 patients. For 3 patients (patient numbers 3, 7, and 8), an AVF was identified by DSA and MRA, but not by CTA.

**Table 3:**
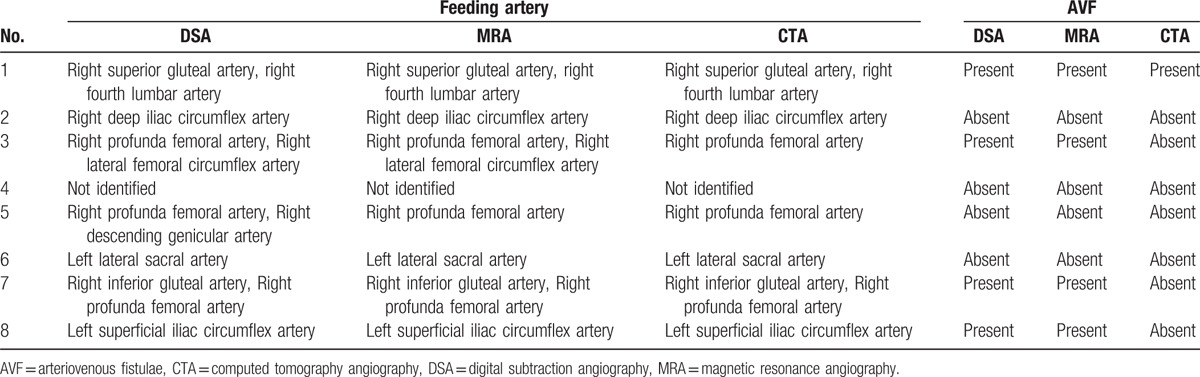
Identification of tumor feeding vessels and the presence of AVF by DSA, MRA, and CTA.

## 4 Discussion

The sensitivity and specificity of MRA and CTA have been reported as comparable to that of DSA for the diagnosis of vasculopathy^[18,19]^; however, data regarding comparing these noninvasive techniques for preoperative assessment of lower extremity tumors are lacking. In the present study, high spatial resolution gadobutrol TWIST MRA with a temporal resolution of 3.84/frame at 3T was evaluated for this purpose. With advances in MR hardware and software, time-resolved MRA can now be obtained with concurrently high spatial and temporal resolution.^[20–22]^ The spatial resolutions utilized in this work were 1 mm isotropic for MRA and 1 mm × 1 mm × 0.625 mm for CTA. Time-resolved CTA was not performed because of the current prohibitively high level of ionizing radiation exposure required.^[8,23]^ The main finding of the present study is that TWIST MRA is comparable to CTA in identifying feeding arteries and AVF and assessing arterial involvement.

Selection of contrast agent is important for contrast-enhanced MRA,^[24]^ including TWIST, because the concentration and relaxivities of the contrast agent affect image quality. Gadobutrol has a high relaxivity and is the only gadolinium (Gd)-based contrast agent approved for clinical use at 1 mol/L concentration. A more compact bolus shape was observed by Dariusch et al^[25]^ after administration of gadobutrol compared with gadopentetate dimeglumine in mini pigs, and overall image quality was rated higher in examinations with gadobutrol. Morelli et al^[26]^ found that gadoburol contrast-enhanced MRA resulted in improved accuracy of renal artery stenosis assessments relative to gadoterate meglumine (Gd-DOTA). Thus, gadobutrol was chosen as the contrast agent for MRA in the present study.

In the present study, image quality of most cases of CTA MIP (without data subtraction) was excellent. Image quality of MRA was slightly lower than that of CTA, with the difference not statistically significant (*P* = 0.135). One possible explanation is that MRA images relied upon mask subtraction, which was susceptible to patient motion. Fortunately, the lower extremity is not affected by respiration or bowel peristalsis like the chest and abdomen; thus, decreased image quality secondary to motion was relatively infrequent. Venous contamination was not observed in the arterial phase of TWIST MRA in this study, which also contributed to the image quality of MRA. Bolus-chase contrast-enhanced MRA is an alternative technique to TWIST in lower extremity MRA, but was not utilized in this study because of the potential for venous contamination.^[27,28]^

The spatial resolution of MRA (1 mm × 1 mm × 1 mm) utilized in this study was sufficient for detection of small feeding arteries. The isotropic resolution also enables rotation of the MIP MRA images out of the coronal plane without significant vessel blurring, which is important for accurate identification of the feeding artery. Although the spatial resolution of CTA (1 mm × 1 mm × 0.625 mm) was even higher, CTA did not identify more feeding arteries than MRA.

Arterial invasion is an issue of particular concern to orthopedic oncologists, for which excellent intramodality agreement (kappa more than 0.8 for both readers) was observed between CTA and MRA in the present study. Thus, with its lack of ionizing radiation, MRA seems an ideal substitute for CTA in the assessment of invasion of the arterial vasculature. However, it is worth noting that the TWIST is performed in the coronal plane, whereas CTA is performed in the transverse plane. The detection of mild anterior-posterior stenosis with MRA was slightly limited, thus underestimating vascular involvement relative to CTA.

Feeding arteries and arterio-venous fistula are usually of very small size, limiting their angiographic demonstration in some cases.^[29,30]^ With the development of more technically sophisticated MR hardware and software, spatial resolution and SNR have also improved. This is likely contributory to the ability of detecting feeding arteries. Furthermore, the conventional CTA had only 1 frame of imaging, whereas the TWIST MRA contained multiple arterial and venous phases, which enable readers to view the dataset dynamically. Similar to DSA, and also to examine the best arterial phase of TWIST to evaluate for feeding arteries. The dynamic nature of the TWIST dataset also enabled to improve identification of AVF, whereas single-phase CTA may fail to detect slow-flow AVF.

One limitation of the present study is that only 1 MR contrast agent was utilized for evaluation versus CTA. Additional data acquired by using other agents is needed to validate the current results. Ideally, DSA would be available for comparison in every patient; however, this is not realistic in practice, from an ethical perspective given the invasiveness of the technique. As a result, only 8 patients underwent preoperative DSA. Nevertheless, TWIST MRA may prove useful for preoperative planning and also for feeding vessel identification before DSA, allowing a more selective approach and efficient procedure. The lower extremity tumors studied by authors included primary soft tissue tumors and primary bone tumors, malignant tumors, and benign tumors. It may be interesting to compare feeding arteries and AVF between them (soft vs. bone, malignant vs. benign) in further study.

## 5 Conclusion

Gadobutrol TWIST MRA is a reliable imaging modality for the assessment of lower extremity tumors and is comparable to CTA for the identification of AVF and feeding arteries, proving an adequate substitute for DSA in many cases.
